# Exosomes and SARS-CoV-2 infection

**DOI:** 10.3389/fimmu.2024.1467109

**Published:** 2024-11-26

**Authors:** Liuying Li, Zixuan Yang, Jia Li

**Affiliations:** ^1^ Department of Traditional Chinese Medicine, Zigong First People’s Hospital, Zigong, China; ^2^ Hospital of Chengdu University of Traditional Chinese Medicine, Chengdu, China; ^3^ College of Medical Technology, Chengdu University of Traditional Chinese Medicine, Chengdu, China

**Keywords:** exosomes, SARS-CoV-2, COVID-19, interplay, immunoregulation

## Abstract

Exosomes, which are small extracellular vesicles, are of particular interest in studies on SARS-CoV-2 infection because of their crucial role in intercellular communication. These vesicles are released by several cell types and are rich in “cargo” such as proteins, lipids, and nucleic acids, which are vital for regulating immune response and viral pathogenesis. Exosomes have been reported to be involved in viral transmission, immune escape mechanisms, and illness development in SARS-CoV-2 infection. This review examines the current research on the contribution of exosomes to the interplay between the virus and host cells, highlighting their potential as diagnostic biomarkers and therapeutic targets in combating COVID-19.

## 1 Introduction

### 1.1 SARS-CoV-2 and COVID-19

Severe acute respiratory syndrome coronavirus 2 (SARS-CoV-2) is a novel coronavirus that emerged in late 2019 and caused a global pandemic (1). SARS-CoV-2 is a member of the Betacoronavirus genus and an RNA virus with a single-stranded genome (2). Its morphological structure comprises four major proteins, namely, nucleocapsid (N), membrane (M), envelope (E), and distinctive spike (S) proteins (3). The S protein, which gives the virus its characteristic appearance, plays a vital role in its interaction with the host cell (3). The S protein comprises two subunits, S1 and S2. The S1 subunit houses the receptor binding domain (RBD), which binds specifically to the angiotensin-converting enzyme 2 (ACE2) receptor on the surface of host cells (4). Upon binding, the S2 subunit facilitates membrane fusion between the viral and host cell membranes, which enables viral entry and replication within the cell. Thus, SARS-CoV-2 exploits the ACE2 receptor as its primary gateway for infection (3, 4). This virus belongs to the coronavirus family, causes respiratory symptoms, and is primarily transmitted via respiratory droplets (5).

### 1.2 Discovery of exosomes and milestones in its research

In 1985, R M. Johnstone’s research team studied sheep reticulocyte vesicle secretion under an electron microscope and observed certain structures in the supernatant of sheep red blood cells cultured *in vitro* (6). Subsequently, in 1989, they were duly designated as exosomes (7). In 1996, a study by G Raposo et al. revealed that immune cells resembling B lymphocytes possess the capability to secrete antigen-presenting exosomes (8). Subsequently, in 2007, H Valadi et al. discovered that exosomes contain mRNA and microRNA, which are transferred to and translated into recipient cells (9). In recognition of their groundbreaking research on membrane vesicle transport, three American scientists, namely, Thomas Sudhof, James Rothman, and Randy Schekman were honored with the Nobel Prize in Physiology and Medicine in 2013 (10). Since then, exosomes have gradually gained attention and become a hotspot in biomedical research. Exosomes have now been reported to play a role in diverse processes such as immune response, viral pathogenicity, pregnancy, cardiovascular disease, central nervous system-related diseases, and cancer progression (11).

### 1.3 Characteristics of exosomes

Extracellular vesicles (EVs) are tiny membrane-bound particles released into the extracellular space when the plasma membrane fuses with multivesicular bodies (MVBs) formed via endocytosis. This process occurs under both physiological and pathological conditions. These vesicles are categorized into three distinct classes based on their size: apoptotic bodies (>1000 nm), microvesicles (100–1000 nm), and exosomes (30–100 nm) (11). These vesicles can travel throughout the body via various body fluids such as blood, urine, and saliva (12). Their diverse cargo enables exosomes to facilitate intercellular communication and modulate several cellular functions (11). Exosomes, which originate from different cell types, encapsulate a wide array of cellular components, including DNA, RNA, lipids, metabolites, cytoplasmic proteins, and cell surface proteins. These cell-derived vesicles play a pertinent role in diversified physiological conditions, such as cancer, inflammation, and infection (13).

## 2 The role of exosomes in SARS-CoV-2 infection

### 2.1 Emerging roles of exosomes during SARS-CoV-2 infection

In recent years, exosomes have garnered widespread scientific attention as key mediators of intercellular communication. Exosome biogenesis shares similarities with virus biogenesis, and the cargo carried by these vesicles can considerably influence virus propagation, dissemination, and infection dynamics (13–15). Emerging studies have observed that exosomes may play critical roles in SARS-CoV-2 pathogenesis. Infected host cells release exosomes that contain viral RNA, proteins, and other bioactive molecules, which potentially facilitate viral transmission and modulate host immune responses (15). Conversely, exosomes exert complex effects on SARS-CoV-2 pathogenesis and either exacerbate or suppress disease progression. In addition, exosomes hold promise as noninvasive diagnostic biomarkers and therapeutic delivery vehicles loaded with biomolecules or drugs (13, 16). The different types of exosomes and their respective functions are presented in **Table 1**. Comprehending the pivotal role of exosomes in viral infections and applying this knowledge to diagnostic and therapeutic strategies may provide beneficial insights into patient prognosis, disease prevention, and the development of novel therapeutic approaches.

**Table 1:** Different kinds of exosomes and their functions.

Sources of exosomes	Cargo	Function	Ref.
SARS-CoV-2 Spike transfected HEK-293T cells	miR-148a and miR-590	MiR-148a blocks USP33 and miR-590 blocks IRF9	(65)
Ginger exosome-like nanoparticle (GELN).	aly-miR396a-5p	GELN miRNAs inhibit S and NSP12 expression; GELN aly-miR396a-5p inhibits NF-κB-mediated inflammation and apoptosis after exosome injection	(26)
Mesenchymal stem cells	miR-146a	MiR-146a via exosomes augment IL-1β	(66)
ACE2-expressing human lung spheroid cells	N/A	Blocks the interaction of SARS-CoV-2 with host cells	(67)
Plasma of MILD COVID-19 patients	N/A	Activate CD4^+^ T helper cells and induce IL-2 secretion	(27)
HEK293T cells treated with IFN-α and IFN-β	N/A	Exosomal hACE2 can specifically block the cell entry and replication of SARS-CoV-2	(24)
Derived from ACE2-overexpressing HEK293 cells	N/A	Block cell entry of multiple pseudotyped SARS-CoV-2 variants including alpha, beta, kappa, lambda, and omicron subvariant	(22)
Exos from COVID‐19 plasma	Tenascin‐c and fibrinogen‐beta	Induce NLRP3 inflammasome, Caspase‐1 and IL‐1β	(68)

CPE, Cytopathic effect; MSC-Exo, Mesenchymal stem cell-derived exosomes; MMP-9, Matrix metalloprotease 9; LSC-Exo, Lung spheroid cells-derived exosomes; USP33, Ubiquitin specific peptidase 33.

### 2.2 Signaling cascades of exosomes

#### 2.2.1 Fusion and formation stage

The biogenesis of exosomes begins with the endocytosis of molecular cargo. Early endosomes are the earliest vesicles formed upon internalization and mark the initial stage of the endosomal trafficking pathway. These early endosomes play a central role in sorting and determining the fate of the endocytosed cargo (17, 18). As these endosomes mature, changes occur in their membrane composition. Over time, the endosomal membrane invaginates and forms smaller vesicles called intraluminal vesicles (ILVs). Multivesicular bodies (MVBs) are structures that contain multiple ILVs enclosed by a membrane. The contents of ILVs are degraded when the MVBs fuse with lysosomes. Alternatively, the fusion of MVBs with the cell’s plasma membrane results in the secretion of ILVs into the extracellular environment, where they are transformed into exosomes (18).

### 2.3 Trafficking process and release

**Figure 1:**
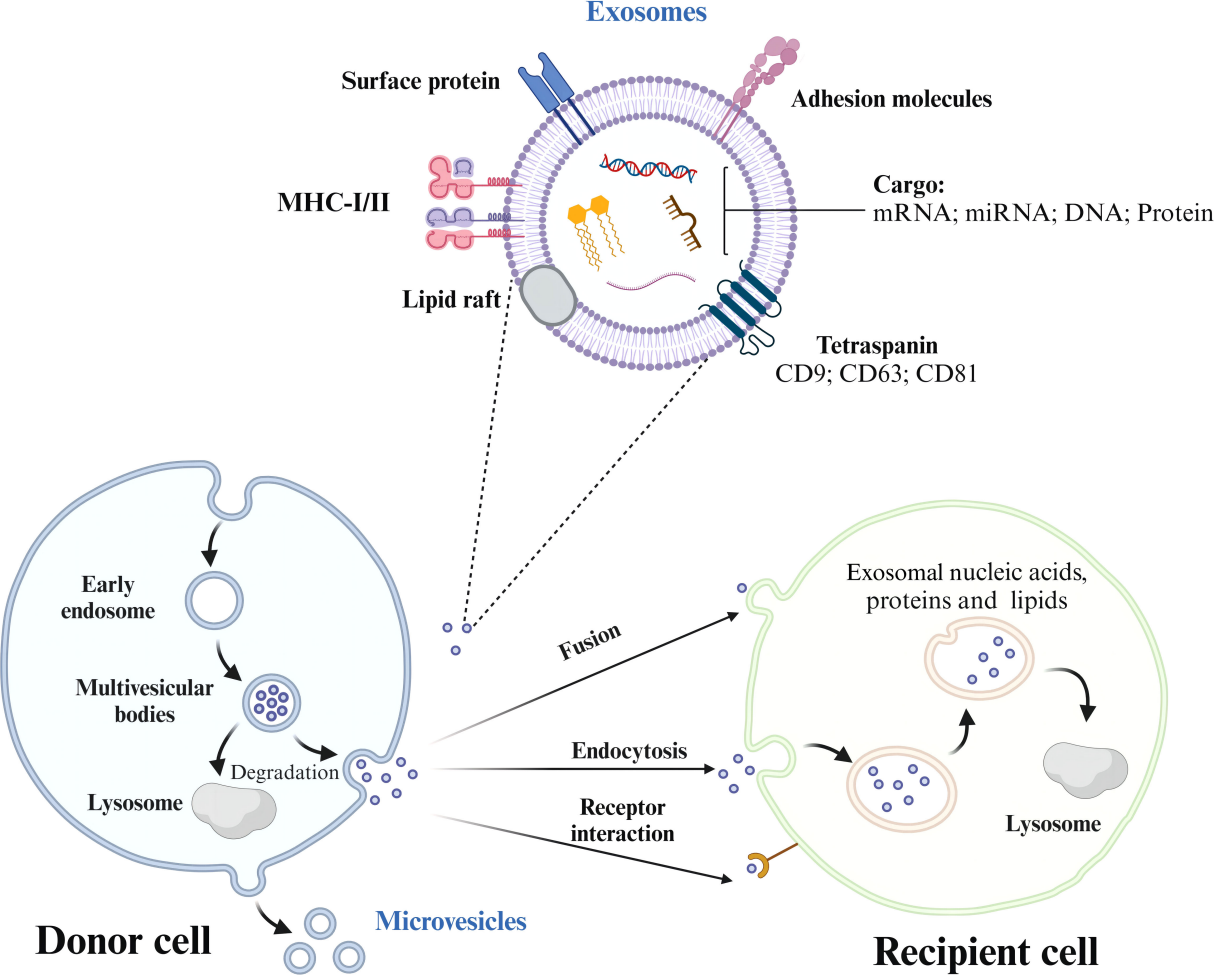
The process of exosome formation, release, and uptake by recipient cells. Exosomes are formed from early endosomes, which ingest molecular cargo via endocytosis. As these early endosomes mature, they develop into multivesicular bodies (MVBs) that contain intraluminal vesicles (ILVs). Depending on their fate, MVBs either fuse with lysosomes to degrade their cargo or with the plasma membrane, releasing ILVs as exosomes into the extracellular space. Exosome trafficking involves the migration of MVBs to the plasma membrane, followed by exocytosis, enabling bioactive compounds to interact with receptors on the target cell.

The cargo has three potential routes from the early endosome. The cargo meant for recycling moves to the peripheral tubular regions of the endosomes and subsequently dissociates and merges either with the Golgi network or the plasma membrane within the recycling endosome. However, the cargo not destined for recycling accumulates in the central vacuolar regions of the early endosome. This accumulated cargo initiates endosomal maturation and leads to the formation of the late endosome. Late endosomes have two possible outcomes: fusion with lysosomes for degradation or fusion with the plasma membrane for exosome release (19). When the MVBs are appropriately stimulated, they migrate from the perinuclear cytoplasm to the plasma membrane and reside in a quiescent state within cells. The MVBs are then fused via exocytosis (**Figure 1**, **Table 2**). The sites of fusion vary depending on the cell type and range from the entire plasma membrane to localized areas. Exocytosis is followed by MVB secretion, which releases the exosomes into the extracellular fluid.

**Table 2:** The main exosome surface protein and cargo, SARS-CoV-2 surface antigen.

Surface protein and cargo			Ref.
**Exosomes**	Surface protein	Tetraspanins (CD9, CD63, CD81, CD37, CD68, CD82, MHC-I, MHC-II)	(69, 70)
	Cargo	Proteins: HSP70/90, Rab	(71–75)
		DNA: mtDNA, cfDNA	(76, 77)
		RNA: miRNA, tRNA, mRNA, rRNA, circRNA, lncRNA, snoRNA	(78, 79)
		Lipids	(80)
		Metabolites	(11)
**SARS-CoV-2**	Surface antigens	Nuclear protein (N), Membrane protein (M), Envelope protein (E), Spike protein (S), NSP1-16	(3, 13, 27)

HSP, Heat shock protein; MHC, Major histocompatibility complex.

### 2.4 Uptake of exosomes by the recipient cells

After the vesicles are released, their membranes undergo predominant activities. Initially, the vesicles face environmental alterations during their transition from the cell’s cytosol to the extracellular fluid. Subsequently, the vesicles interact with the plasma and endocytic membranes in the target cells. Ultimately, the ensuing merging of EVs with cellular membranes marks the end of the extracellular EV route. When liberated from progenitor cells, some vesicles remain intact for a brief period before undergoing membrane disintegration. These vesicles release bioactive substances such as interleukin-1β and various growth factors (TGFβ, FGF, VEGF, etc.), which allows them to bind directly to receptors on neighboring cells and initiate specific reactions. Nonetheless, most EVs resist membrane degradation and remain in the extracellular fluid for extended periods. Surface enzymes and various molecules facilitate pre-binding interactions with adjacent cells, which contribute to the breakdown of extracellular structures. Furthermore, EVs often accumulate near intercellular junctions in the extracellular areas and traverse the intercellular space. As a result of these movements, EVs exit the original fluid and migrate to nearby tissue regions (20), potentially entering larger fluid bodies, such as the blood serum, lymph, and cerebrospinal fluid. Once the target cells are identified, EVs (often studied using optical tweezers) establish connections with their surface (21). Finally, the vesicles merge with the plasma or endocytic membrane and release the luminal contents into the cytosol.

## 3 Exosomes regulate viral infection via various molecular mechanisms

### 3.1 Exosomes containing ACE2 proteins bind competitively to viral S protein

**Figure 2:**
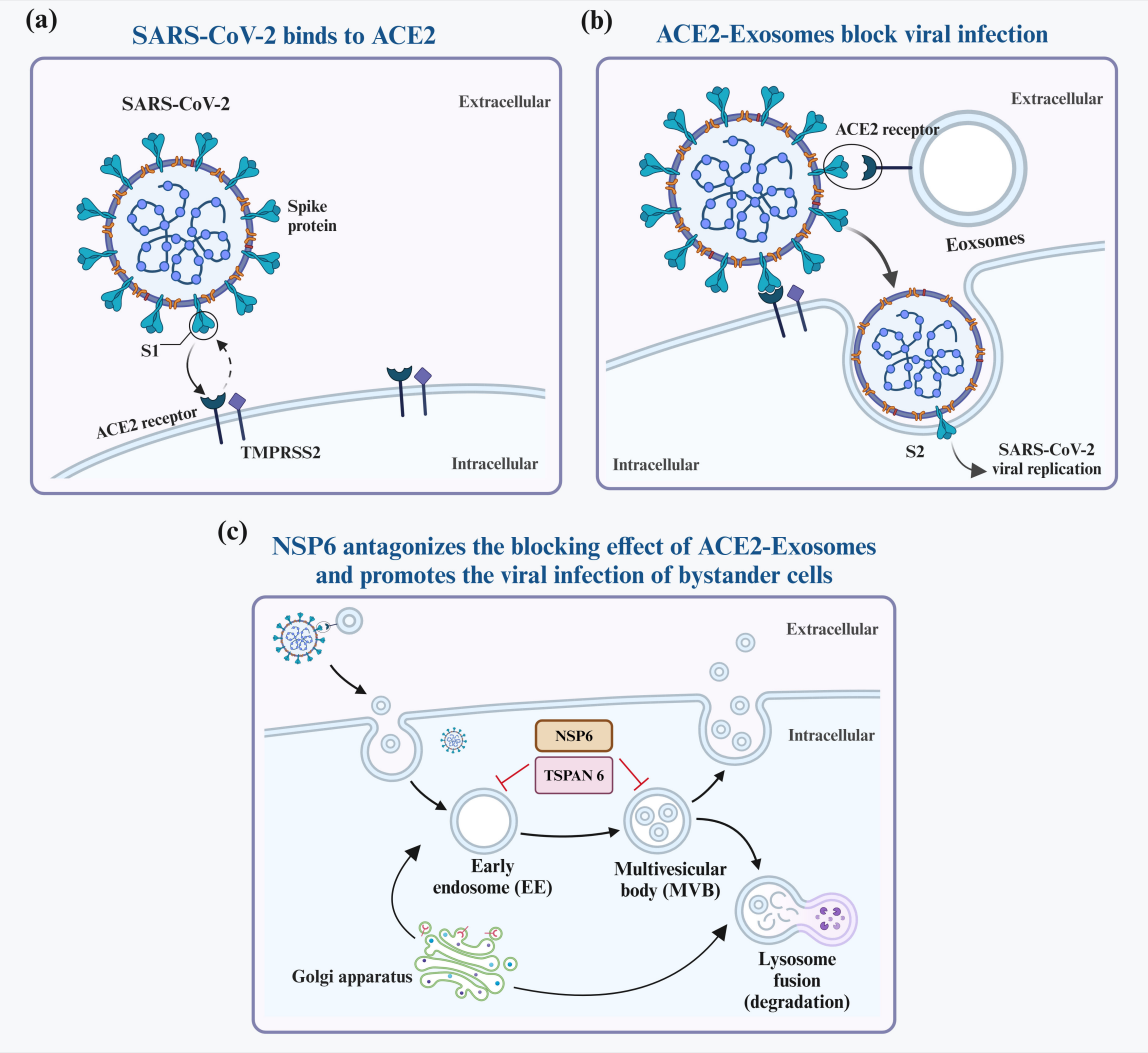
Interaction between exosomes and SARS-CoV-2. SARS-CoV-2 infects host cells predominantly by attaching to ACE2 receptors on their surfaces **(A)**. To block infection, ACE2-containing exosomes (ACE2-Exosomes) can be activated and bind to free virions, preventing susceptible bystander cells from becoming infected **(B)**. However, SARS-CoV-2 NSP6 suppresses the formation of ACE2-Exosomes, thereby enabling viral infection of neighboring cells **(C)**.

Elevated levels of EVs expressing ACE2 (evACE2) in the blood of patients with COVID-19 are marked by distinct exosome markers (22). These vesicles can neutralize SARS-CoV-2 by competitively binding to ACE2. Lv et al. reported that SARS-CoV-2 nonstructural protein 6 (NSP6) could suppress the antiviral action of ACE2-exos and promote viral invasion. Furthermore, tetraspanin-6 negatively regulates exosome production (**Figure 2**) (22, 23). In human ACE2 (hACE2) mice infected with SARS-CoV-2, the presence of evACE2 has been linked to reduced mortality rates (21). evACE2 inhibits SARS-CoV-2 infection by blocking the binding of the viral S protein with its cellular receptor ACE2 in host cells. Compared with vesicle-free recombinant hACE2, evACE2 demonstrates a 135-fold higher potency in blocking the binding of the viral S protein RBD and a 60–80-fold higher efficacy in preventing infections by both pseudotyped and authentic SARS-CoV-2 (21). These findings suggest a promising therapeutic avenue to manage COVID-19. Furthermore, treatment with IFN-α/β has been noted to augment the expression of hACE2. This exosomal form specifically inhibits viral entry into target cells, thereby suppressing SARS-CoV-2 replication both *in vitro* and *ex vivo* (24). An intranasal SARS-CoV-2 vaccine utilizing EVs derived from *Salmonella typhimurium* has been shown to elicit neutralizing antibodies against both wild-type and Delta variant strains (25). Moreover, the novel SARS-CoV-2 vaccine candidate based on bacterial EVs has been documented to alleviate lung lesions and improve weight loss (25). Compared with animals in the control group, the vaccinated animals experienced significantly less body mass loss after the viral challenge and, in some instances, even showed mass gains (25). In addition, vaccinated hamsters displayed fewer focal patches of inflammation, alveolar collapse, and hemorrhagic areas of the lung. These observations emphasize the significance of EVs in developing SARS-CoV-2 vaccines (25).

### 3.2 Exosomes contain proteins that regulate antiviral immune responses

Exosomes originate from various cells that contain distinct proteins. For example, exosomes released by virus-infected cells carry viral protein particles that facilitate viral spread. A novel biological activity of SARS-CoV-2, i.e., activation of macrophages via the NF-κB-mediated pathway, was identified (26). Lung epithelial cell exosomes deliver NSP12 to macrophages, triggering their activation via NF-κB. Subsequently, the activated macrophages release inflammatory cytokines that lead to lung inflammation (26). In addition, exosomes carrying NSP13 have been observed to synergistically enhance NF-κB activation along with NSP12 (26). Metabolites released from macrophages activated by exosomes NSP12 and NSP13 have been shown to induce apoptosis in lung epithelial cells (27). These findings highlight the role of exosomes in delivering viral proteins and modulating immune responses in lung epithelial cells.

### 3.3 Mechanism of exosome delivery of noncoding RNAs in viral infection

Noncoding RNAs (ncRNAs) play crucial roles in regulating cellular immunity (28). Several types of ncRNAs, such as microRNAs (miRNAs), long noncoding RNAs (lncRNAs), and circular RNAs (circRNAs), influence cell development, proliferation, and metabolism via diverse mechanisms (29, 30). Valadi et al. first reported that exosomes from murine and human mast cell lines (MC/9 and HMC-1) contain miRNAs and mRNAs that can be transferred to other cells (9). Studies have observed that various ncRNAs can be encapsulated and transported by exosomes (31, 32).

Moreover, exosomal mRNAs can be transported to recipient cells, where they are translated and contribute to the recipient cell’s protein expression. For instance, when internalized by TCA8113 cells, full-length ECRG4 mRNA occurring in serum exosomes inhibits receptor cell inflammation, angiogenesis, and cell proliferation (33). The presence of miRNAs within exosomes implies that they can be directly transported to specific cells and functionally influence mRNA targets. In the context of disease mechanisms, exosomes derived from vascular smooth muscle cells enable the transfer of miR-155 induced by KLF5 from smooth muscle cells to endothelial cells. This transfer promotes endothelial injury and atherosclerosis progression by suppressing the expression of zonula occludens-1 (34). In addition, specific proteins in lncRNA vectors regulate the sorting of lncRNAs into exosomes, and lncRNA–RNA–binding protein complexes selectively collect and sort specific miRNAs (35). Similarly, circRNAs, like lncRNAs, can be transported by exosomes between donor and recipient cells. MCPyV circALTOs are enriched in exosomes derived from VP-MCC lines and circALTO-transfected 293T cells. Also, purified exosomes can mediate ALTO expression and transcriptional activation in MCPyV-negative cells (36).

### 3.4 Mechanisms involved in exosomes transporting other cargo to orchestrate immune responses

Exosomes possess unique abilities to target specific tissues or cells and traverse biological barriers, including the blood–brain barrier, which makes them promising candidates for targeted drug delivery. These vesicles can effectively transport a wide array of therapeutic substances, which range from genetic drugs to traditional Chinese and Western medicines (37, 38). Owing to these inherent advantages, exosomes are versatile vehicles for precise and efficient drug delivery. For instance, paclitaxel (PTX), the drug used extensively in cancer treatment, faces challenges such as high hydrophobicity, dose-dependent toxicity, and side effects (39, 40). In a recent study by Wang et al., exosomes derived from classically activated M1 macrophages were used as carriers to mitigate PTX toxicity and improve its bioavailability. PTX was successfully delivered to tumor tissues in mice, inducing a proinflammatory response via NF-κB pathway activation, thereby augmenting the therapeutic efficacy of the drug (41). Catalase (CAT), a potent antioxidant used to treat neurodegenerative diseases by inhibiting inflammation and protecting dopaminergic neurons, is limited by the impermeability of the blood–brain barrier to most therapeutic agents (42, 43). Haney et al. found that coating the enzyme with exosomes effectively reduces oxidative stress and enhances neuronal survival in both *in vivo* and *in vitro* models. Loading CAT onto exosomes preserves its biological activity, extends its blood circulation time, decreases its immunogenicity, and overcomes issues such as rapid degradation, considerably improving the therapeutic efficacy (44).

## 4 Clinical findings and correlation between exosomes and disease severity

### 4.1 Association between exosomes and disease severity

Serum-derived exosomes in patients with COVID-19 have been linked to disease severity (27). Elisa et al. analyzed plasma samples from 20 patients with SARS-CoV-2 infection and observed that those with mild symptoms had a higher number of circulating SARS-CoV-2-S exosomes than those with severe symptoms (27). In another study, Song et al. reported elevated levels of gangliosides and sphingomyelin in the serum of patients infected with SARS-CoV-2, along with the absence of diacylglycerol, which is a distinct lipid pattern specific to exosomes (45). In their study, Kwon et al. showed that pulmonary epithelial A549 cells transfected with nonstructural and structural genes of SARS-CoV-2 released viral RNA-rich exosomes (46). Hence, analyzing SARS-CoV-2-related markers within exosomes isolated from the patient’s body fluids can aid in evaluating the viral replication status and immune response, facilitating predictions of disease severity and prognosis (47–49). Several studies have observed that infection with SARS-CoV-2 results in a general increase in the expressions of human endogenous retroviruses (HERVs) and immune response mediators (50, 51). Endogenous retrovirus transcripts and proteins can be exported in EVs, which makes HERVs a contributing element in COVID-19 and early genomic biomarkers to predict COVID-19 severity and outcome (52, 53). Thus, SARS-CoV-2 exosomes are vital indicators of the functional status of the patient’s immune cells and exhibit distinct characteristics in those with mild symptoms (27). This unique feature provides a theoretical foundation for further studies on alternative exosomal approaches for preventive or therapeutic strategies against SARS-CoV-2 infection.

### 4.2 The application of exosome-based vaccines in clinical diseases

Exosomes have emerged as a promising therapeutic option owing to their advantageous characteristics, such as small size, non-toxicity, low immunogenicity, high stability, and ease of storage (54). In the field of vaccine development, exosomes are being actively investigated as a platform to construct safe and efficacious vaccine vectors. For example, exosomes loaded with the SARS S protein can effectively induce neutralizing antibody (55). A chimeric protein (SGTM) was engineered in the study by replacing the transmembrane domain of SARS-S with the G protein of the vesicular stomatitis virus, which resulted in a vaccine against the SARS coronavirus (55). This innovative exosome-based vaccine strategy can overcome the challenges linked to conventional vaccines, such as storage and stability issues. Exosomes can protect their cargo from degradation and may evade neutralization by antibodies, which enhances the efficacy and durability of the vaccine (56, 57). Moreover, engineered EVs expressing the ACE2 receptor can act as decoys to prevent the SARS-CoV-2 S protein from infecting healthy cells (58). A study showed that EVs expressing ACE2 and TMPRSS2 decreased the infection rate of healthy Caco-2 cells by 50% (59). Another study proposed the use of evACE2 derived from mesenchymal stem cells to bind the S protein competitively. This binding protected the cells from damage and maintained ACE2 surface expression. Acute lung injury and endothelial dysfunction caused by SARS-CoV-2 infection were thus prevented (60).

## 5 Conclusion

Exosomes are crucial vectors for virus transmission and influence viral replication in two ways. On the one hand, they promote virus replication, transmission, and infection and downregulate antiviral immunity (14, 61, 62). On the other hand, they limit viral infection and potentiate antiviral immunity. For instance, HIV packages viral proteins and RNA into vesicles during cellular infection. These vesicles are later released into the extracellular space, which aids in viral spread to noninfected cells (63). Nevertheless, this phenomenon has not yet been demonstrated in SARS-CoV-2 (64).

EVs expressing ACE2 neutralize SARS-CoV-2 by competitively binding to ACE2 and specifically blocking viral entry into target cells, thereby inhibiting viral replication *in vitro* and *ex vivo*. The studies conducted so far have confirmed that exosomes are a double-edged sword in viral infection. Future studies should aim to elucidate these dual roles and examine how exosome-mediated interactions can be harnessed to devise novel therapeutic strategies against COVID-19. Understanding the specific mechanisms by which exosomes influence viral behavior and immune responses may provide beneficial insights for designing effective interventions. Moreover, investigating the potential of exosome-based therapies could open new avenues to enhance antiviral immunity, paving the way for innovative therapeutic strategies that leverage the natural properties of exosomes in combating viral infections.

In summary, the nuanced roles of exosomes in viral transmission and immune modulation present challenges as well as opportunities for future research. The continued exploration in this field may not only broaden our understanding of viral pathogenesis but also contribute to the development of more effective antiviral therapies.

## 6 Outlook

Despite the promising potential of exosomes in treating COVID-19, research in this area is fraught with several challenges. Exosome purity and safety should be ensured, particularly for clinical applications, and protocols for mass production, transport, storage, management, and monitoring of exosomes should be standardized. A lack of these measures may hinder the transition from laboratory research to clinical implementation. Moreover, current research has not clarified the mechanisms by which exosomes operate in the SARS-CoV-2 infection process. Several issues warrant further investigation, including how exosomes specifically influence viral transmission and immune responses and the ways to optimize their application for enhancing therapeutic efficacy. Exploring the important roles and unique characteristics of exosomes in the human body could, therefore, improve the effectiveness of immunotherapy for COVID-19, bringing us one step closer to clinical implementation in the future. A wealth of basic research in exosome delivery systems suggests that they can help overcome the challenges and dilemmas associated with the treatment of SARS-CoV-2 infection. By leveraging these insights, we can develop innovative therapeutic strategies that harness the potential of exosomes in combating COVID-19.
